# Development and validation of the shared governance feasibility instrument in nursing schools in Iran

**DOI:** 10.1186/s12912-020-00433-x

**Published:** 2020-05-20

**Authors:** Maryam Sattarzadeh-Pashabeig, Foroozan Atashzadeh-Shoorideh, Mohammad-Mehdi Sadoughi, Alice Khachian, Mansoureh Zagheri-Tafreshi, Alessandro Stievano

**Affiliations:** 1 Student Research Committee, School of Nursing and Midwifery, Shahid Beheshti University of Medical Sciences, Tehran, Iran; 2 Department of psychiatric Nursing and Management, School of Nursing and Midwifery, Shahid Beheshti University of Medical Sciences, Vali-Asr Avenue, Cross of Vali-Asr and Hashemi Rafsanjani Highway, Opposite to Rajaee Heart Hospital, Tehran, 1996835119 Iran; 3 Department of Ophthalmology, School of Medicine, Shahid Labbafinezhad Hospital, Shahid Beheshti University of Medical Sciences, Tehran, Iran; 4 School of Nursing and Midwifery, Department of Medical Surgical Nursing, Iran University of Medical Science, Tehran, Iran; 5 Center of Excellence for Nursing Scholarship OPI Rome, Rome, Italy

**Keywords:** Nursing education, Nursing schools, Psychometrics, Shared governance

## Abstract

**Abstract:**

To develop and validate a shared governance feasibility instrument in schools of nursing in Iran with respect to the nature of the profession and the sociocultural context of the Iranian community.

**Background:**

Nursing schools are liable to the application of shared governance due to the presence of various expert educational groups within the school that necessitates reciprocal cooperation. Since the concept of shared governance is culture-based and given that no full-fledged study has been conducted on shared governance in Iran, the development of a suitable shared governance feasibility instrument is rendered as mandatory.

**Methods:**

This sequential exploratory mixed-method study consisted of two qualitative and quantitative parts was accomplished 2016–2019. First, the primary items were extracted through an extensive review of the literature, qualitative interviews and underwent psychometric validation using a methodological approach. Face, content, construct validity and reliability of the instrument was established and completed.

**Results:**

One hundred fifty items were distilled from the first stage of the study, was reduced to 70 after establishing face, content validity and primary reliability. Exploratory factor analysis resulted in 52 items covering the two factors “shared atmosphere and culture” and “infrastructural prerequisites”. These two factors accounted for 78.6% of the total variance of the questionnaire. In calculating the final reliability coefficient of the instrument, Cronbach’s alpha and Omega were 0.981 and 0.805, respectively. The results showed an ICC of 0.91 indicating high reliability of the developed instrument with a standard error of measurement (SEM) of 10.43. Finally, the items underwent weighting via scoring by considering item weights due to differences between the two methods.

**Conclusion:**

“Shared governance feasibility instrument” can provide a new insight into organisational performance for all policy-makers and beneficiaries of higher education. This not only leads to the use of intelligence and capabilities of the beneficiaries, but also aids in faster movement toward achieving organisational goals.

**Implications for nursing management:**

This study and the developed instrument may serve as a guide for the feasibility of implementing shared governance to assess management styles and performance in higher education centers.

## Background

Shared governance is a structure in which the individuals of one organisation to undertake the decision-making process. This allows them to take accountability, responsibility, and ownership of decisions. Although shared governance is typically referenced regarding nursing practice, it can be adapted to higher education [1]. Definition of shared governance is difficult because it is utilised differently within each organisation [2].

Organisations that create a participatory environment for staff are more likely to support a shared vision and a sense of empowerment among team members [3]. A sense of belonging can create a culture in which employees are more engaged in their work. Organisational cultures with high levels of engagement and satisfaction can expect greater productivity and retain more motivated employees [2]. In spite of these advantages, schools and universities are often inefficient at creating engagement opportunities among faculty and staff [4].

Iranian higher education institutes have expectations of their faculty members, but they have not yet clearly defined the faculty roles. The professional roles for a faculty in universities are generally three parts such as teaching, research, and executive service. In recent years, the Ministry of Health and Medical Education added a cultural-educational-social role [5]. These changes have enhanced the importance of effective internal governance in such institutes [6]. Shared governance has been one of the important management strategies for many years in other disciplines have benefited from the application of these principles such as commerce, education, political sciences, and religious sciences [7].

Cleland (1978) introduced the shared governance model into the nursing literature, with the concept of an academic model of governance by faculty members. This model incorporated the interests of all groups in organisational policy-making, by the distribution of power among different groups in the organisation [8]. Although, shared governance has been utilised in many countries such as America [9], England [10], Pakistan [11], China [12], Jordan [13] and Jamaica [14], the models and indicators of shared governance were criticized many times [3, 4, 11, 15]. In addition, no suitable instrument has been found by authors to measure the feasibility of shared governance at universities around the world.

Shared governance is a strategy that assigns faculty members a position similar to that of managers, enabling them to take part in decision-making that, in turn influences managers’ performance [13]. The common characteristics of various definitions of shared governance are lack of dependence in performance, responsibility, empowerment, development, contribution, and collaboration in decision-making. The process by which these goals are reached may differ considerably among organisations [16, 17].

Shared governance in nursing is a way of providing individuals with a position for decision-making in their performance, like that of managers [8]. The concept of shared governance has been defined in a study in Iran as being like “several souls in one body” that not only shares the above-mentioned characteristics, but has also considered spirituality in the organisations as a feature of such shared governance [13, 17].

Nursing education predisposes to training professional nurses who can use this capacity to investigate and recognise the health status of people who can and will provide care in different fields to people, families, and communities [8]. Decision-making in nursing schools and many higher education centers is commonly performed in a hierarchical order that is the antithesis of shared decision making [16]. In Iran, faculty members seldom have role in policy-making and decision-making [13]. For greater efficiency, it would seem appropriate that State universities should move their governance structure towards shared responsibilities [11].

Most shared governance models implemented in education centers worldwide are based on seven indicators introduced by the seminal Ramo’s shared governance model. These represent the institutional climate for governance, institutional communication, joint decision-making, the role of the institutional governing board, the role of the institutional president, the role of the faculty at the institution and assessing the structural arrangement for governance [18].

Ramo’s model may not be applicable everywhere, and the existence of different cultures in different countries has brought about variable outcomes in implementation of shared governance [14]. Hence, it appears that to implement shared governance, it is necessary to first carry out investigations based on the organisational culture governing any organisation to determine accurately the concept and the structure of shared governance [8]. An instrument named the “American Association of University Professors Indicators of Sound Governance” (AAUPISG) was introduced on the basis of Ramo’s indicators of shared governance to investigate the rate of correspondence between the performance of higher education centers and the national standards of shared governance [19].

Most studies conducted on shared governance have either not used specialized shared governance instruments [13], or utilised the AAUP indicators in its original or adjusted forms [12, 20]. In some cases, only some part of the above-mentioned instrument has been used [11]. Additionally, reviewing the literature was found that no fully-fledged study explored or reported the feasibility of implementation of shared governance in Iranian educational centers [7, 21].

As the concept of shared governance has not been widely recognised in Iran and elsewhere, the investigation of whether this is a feasible construct, but also an instrument with demand to be developed in Iran. In the present study, which is part of a larger scale study on the concept of shared governance in the sociocultural contexts of the Iranian community, we have developed and validated psychometrically suitable instrument for assessing the feasibility of implementation of shared governance as a construct in nursing schools affiliated to three major medical universities in, Tehran. The aim of this study is developing and validating a shared governance feasibility instrument.

## Methods

### Design

This is a sequential exploratory mixed-method study, conducted from 2016 to 2019. In this research, tool development consisted of two qualitative and quantitative stages initiated with the qualitative stage and followed by quantitative follow-up.

The qualitative stage includes two phases. Phase 1 (hybrid phase) was completed with three steps: literature review (first step), the field work (second step) and integration of codes emerged from steps 1 and 2 (third step). Phase 2 included item generation based on the first phase.

In the quantitative stage of this research tool validation (face, content validity, exploratory factor analysis, and reliability) was done.

**Fig. 1:**
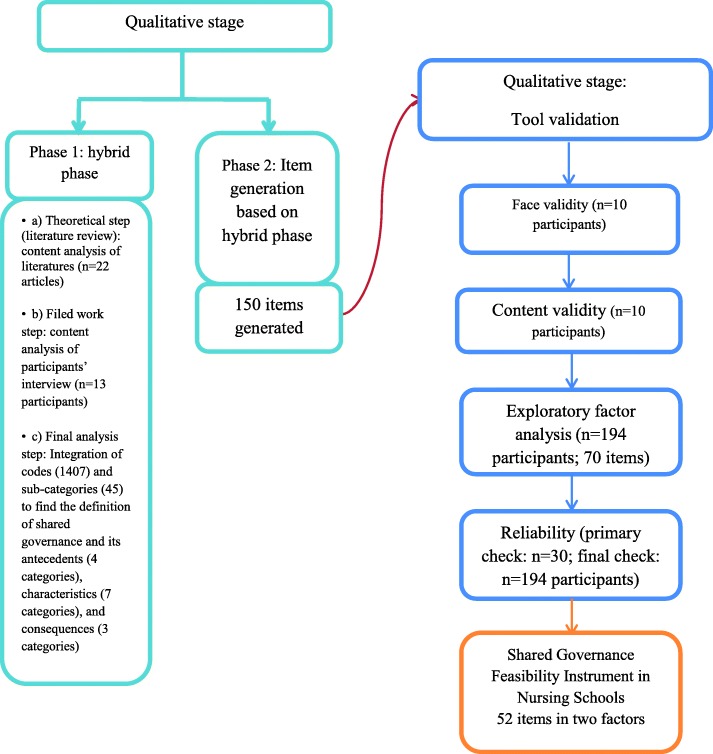
Sequential exploratory design of study

The definition of shared governance, characteristics, antecedents, and consequences were extracted from the results of the qualitative stage (first phase). Using the findings of this phase, the themes were defined and developed into an instrument that was subsequently measured psychometrically using the methodology approach in the second stage. In this quantitative (second) stage of the instrument development, face, content validities, exploratory factor analysis (EFA), and reliability were used (Fig. 1).

### Participants

In the first (theoretical) step of the qualitative stage, 22 articles obtained from a review of the literature were initially analysed. Thereafter, during the fieldwork step, 13 participants including one member of a nursing board in the Ministry of Health (MOH), three intermediary managers of medical universities in Tehran city, and nine faculty members of nursing schools were interviewed. They were selected with a purposive sampling method designed to ensure maximal variation in age, gender, specialty, and work experience in higher education centers [17, 21].

In the quantitative stage (tool validation), 10 managers and faculty members of the nursing schools participated in the face validation, 10 in the content validation, 30 in the primary reliability establishment and 194 faculty members in the construct validation and last reliability processes.

For construct validation, at least three participants were required for each item in the quantitative stage. Two hundred fifty questionnaires were distributed to faculty members of nursing schools five of the biggest or highest schools in Iran’s ranking system selected with a convenient sampling method. The inclusion criteria were voluntary participation and at least two-year work experience as a full-time faculty member in the school. Of these 250 questionnaires, 194 completed questionnaires were returned and these were analysed for construct validation (response rate = 78%).

### Data collection

Concept analysis and determination of the final operational definition of context-based shared governance using hybrid concept analysis was done in the first step [17]. To extract items from the available literature in the theoretical (second) step, the related papers were searched with the keywords “tool/instrument/ questionnaire, faculty member, higher education, shared governance, feasibility, and psychometric” using the databases “Google Scholar, PubMed, Science Direct, and Eric” between 1990 and 2017. Simultaneously, Persian databases like MedLib, Scientific Information Database (SID), Magiran, Iran Doc, and Iran Medex were searched with Persian keywords for shared governance: these searches did not return a single related paper. Of 349 papers retrieved from the initial search, 22 articles related to exact research goals and related to shared governance instruments and guidelines. In the fieldwork step, the first author performed semi-structured deep interviews with 13 participants to extract the intended items [17, 21]. The inclusion criteria consisted of any studies that were in Persian or English language, systematic review, qualitative and/or quantitative, integrative and psychometric, and full-text articles. The studies that did not relate to college or universities were excluded.

After analysing the data obtained from the theoretical and fieldwork step, integrating the findings, extracting codes and categories using the inductive-deductive approach, the items pool, consisting of 150 items including characteristics, antecedents, and consequences of the qualitative stage, entered the quantitative stage.

In the quantitative stage, the instrument underwent quantitative and qualitative face and content validation by 10 faculty members; it then underwent primary reliability establishment and item analysis. Some items had been changed or omitted. The questionnaire was then distributed to faculty members with 70 items.

### Data analysis

In the qualitative stage, data were analysed by the method of Schwartz-Barcott & Kim [22]. Conventional content analysis by Graneheim & Lundman was used to determine the concepts and to extract codes and categories [23]. The full text of each paper in the theoretical step and each interview in the field-work step was considered as an analysis unit [24]. Then, each article was read three times to arrive at an agreed general content. The primary codes were then extracted as explicit and implicit concepts. Then, similar codes (obtained from explicit and implicit concepts) were classified as subcategories, which in turn, were put into a group. The categories were subsequently labeled [23]. In the third step, the data obtained from the theoretical and fieldwork steps were merged and the item pool was obtained.

In the quantitative stage of the study, the methodological approach was applied to determine the psychometric properties of the first version of the instrument with 150 primary items. Having applied the corrections in the qualitative stage, quantitative face validity was established by measuring item impact. To make sure that the items measured the intended construct, content validation was done both quantitatively and qualitatively. To explore CVR, 10 managers and faculty members were asked to assess the necessity of each item. To survey CVI, the managers and faculty members were asked to express their opinions about the rate of relatedness of each item with the intended construct [25].

Regarding raters consensus on item relatedness, the modified Kappa 1 statistic [26] was used; this provides instrument developers with information on the degree of consensus without chance ratio. A corrected Kappa statistic greater than 0.74 was rendered as excellent, between 0.6 and 0.74 as good, and smaller than 0.6 as poor [27]. Colton & Covert (2007) mentioned item analysis (IA) as one way of construct validity assessment; to examine construct validity, the correlation between each item and other items and the whole instrument was performed. To investigate the primary reliability of the instrument before validation, the internal consistency coefficient (Cronbach’s alpha) was used.

In this study, factor analysis in construct validation [25] and exploratory factor analysis (EFA) with maximum likelihood by the use of oblique rotation of the Promax type was utilised to determine the degree to which the developed instrument measures the concept of shared governance. Also, EFA was used to determine the relations among the latent and observed variables and then the significance and severity of these relations [28]. By doing this, the three criteria of Kaiser’s criterion, Scree plot and cumulative variance percentage determined by extracted factors were used. The KMO statistic was estimated to test sample volume sufficiency in which 0.8 and greater were rendered as suitable [28]. Moreover, the scree plot was plotted to determine the number of factors. Plotting of the factors formed and a horizontal line was rendered as a reference [29].

In this study, three samples per item were rendered as sufficient in factor analysis [30]. The study sample consisted of faculty members at nursing schools of major Iranian medical universities selected based on inclusion criteria as mentioned before. The developed instrument was completed via self-report. Considering that the instrument contained 70 items, given the ratio of 3 samples per item, completion of at least 210 questionnaires was necessary [28]. On this basis, considering a probable rate of deficient or unreturned questionnaires, 250 written questionnaires were distributed to faculty members of nursing schools, of which 56 questionnaires were not returned. Hence, 194 completed questionnaires (response rate 78%) were collected. SPSS20 was used in data analysis.

Finally, the reliability of the instrument was established using both internal consistency and stability. To estimate internal consistency, Cronbach’s alpha and conventional odd/even split-half reliability were used. Additionally, test-retest reliability and intra-class correlation coefficient with the 2-week interval was used on 30 faculty members to examine the relative consistency. The estimation of ICC is used to assess the consistency of the intended measured variable by the use of an instrument for similar individuals in two different situations. A 2-week to the 1-month interval between two tests is suitable [25].

In this study, 30 participants were used to determine consistency of the developed instrument so that they completed the final instrument twice with a 2-week interval. Koo & Li have rendered ICC less than 0.5 as weak, between 0.5 and 0.75 as moderate, between 0.75 and 0.90 as good, and greater than 0.90 as excellent consistency [31] . Ultimately, SEM was used to determine the accuracy of test measurement. SEM is one of the indices of measurement accuracy that shows an estimation of acceptable expected deviation from real values in a group of measurements in a specific condition, i.e., SD of scores distribution [25]. Moreover, the weight and significance of each item in each factor were determined by the use of results of factor analysis and loading of each item onto that factor [32].

Scoring of instruments is possible on the basis of Likert rules and/or linear transformation method so that the choices open to each item ranged from very much =5 to very little =1. To determine whether weighting created any changes in the items of the instrument, the mean rank of each item was estimated by paired t-test before and after weighting. The hierarchical position of the item in the instrument was determined in two states based on these mean ranks [32].

## Results

The operational definition of shared governance, characteristics, antecedents, and consequences were extracted from the results of content analysis in qualitative stage of this study. According to the findings, shared governance is a unique multi-lateral concept and an eccentric (non-centered) structural model in which all beneficiary parties with contribution-based relations participate as a unique entity in a contributory milieu via understanding the importance of inter-personal conflicts based on spirituality. In such an environment, they are all responsible for their duties. The prerequisite of such governance is the presence of infrastructural factors, committed managers and faculty members in the unique context of higher education who try their best in line with the needs of the modern era. The result of such an attempt will be the promotion of organisational commitment, personal and organisational development.

Given the definition of shared governance in this study, it may be asserted that in the Iranian cultural context, this concept was very similar to many other countries. However, organisational spirituality was highly emphasized in Iran due to the religious and cultural context of our country and due to the importance of nursing and midwifery professions that deal with human lives.

In the qualitative stage of the study, the results of the theoretical step (22 articles) and the fieldwork step (13 participants) were used to extract the items. There were eight female and five male participants with the youngest being 41 and the oldest being 56 years old. Their work experience ranged from 6 to 29 years. In addition, their managerial experience showed a range from 1 to 24 years. All participants, except for one general practitioner, were specialists in nursing school management [17, 21].

The results of the hybrid phase led to the extraction of 470 primary codes in the theoretical step and 937 primary codes in the fieldwork step. These codes were finally classified in the final analysis step into 45 subcategories and 14 categories based on semantic similarity, and a general theme labeled “several souls in one body” (Table 1) [17, 21].

**Table 1:** Integrated categories and subcategories of theoretical stage and field work

	***Category***	***Subcategories***
**Antecedents**	Participatory context of higher education institutions	− Uniqueness of schools/higher education Centers − Uniqueness of faculty members − Uniqueness of nursing education
	Infrastructural obligations	− Facilitative rules of participation − Corresponding intra-organisational support − Resources suitable for work − Learning the team work
	Coordination with contemporary requirements	− Members’ needs − Managers’ needs − Organisations’ requirements
	Participation-oriented managers	− Managers as symbol of participation − The intrinsic and acquired competencies of the managers − Involving faculty members in school/higher education centers management − Role of chief administrators in promoting and implementing culture of shared governance
**Characteristics**	Participatory Climate and Culture	− Adaptation to change − Common goals − Mutual respect − Equality among stakeholders − Coordination − Mutual trust − Empathy
	Conscious participatory decision-making	− Participatory decision-making − Participatory understanding − Transparent exchange of organisational knowledge
	Mutual accountability	− Accountability of all the stakeholders − Importance of accountability
	Multiplicity of the ideas	− Necessity of conflict − Conflict management
	Decentralized structure	− Participatory structure − Participatory organisational culture − Continuous participation
	Interrelationship	− Communication as a key factor − Open vertical and horizontal communication − Establishment of appropriate formal and informal relations
	Sublime organisation	− Spiritual goals − Promotion of religious ethical values − Following religious guidelines
**Consequences**	Promotion in Organisational Commitment	− Members’ satisfaction − Organisational attachment
	Individual Development	− Faculty members’ autonomy − Blooming the talents
	Organisational Development	− School dynamics − Increasing Productivity − Promotion of the Institute’s ethics and culture − Power distribution

In the second phase (item generation) of qualitative stage, an inductive- deductive method was used to develop 268 primary items on the basis of the results of the theoretical and interview phases using a 5-point Likert scale without reverse scoring ranging from “very little” = 1 to “very much” = 5. After merging the overlapping items by the research team, the instrument entered a qualitative face validation stage with 150 items.

After reviewing the participants’ opinions by the research team in the qualitative face validation stage, 5 out of all items were divided into two separate items and thus a new item was added. In addition, 37 items were omitted due to their great similarity with other items. One item was omitted due to the absence of any part-time faculty members in the nursing schools and another was deleted as it pertained just to faculty clinicians. Next, 6 more items were excluded in the quantitative face validation due to an impact score of less than 1.5. At this stage, the shared governance feasibility instrument entered the content validation stage with 111 items. Given the respondents’ comments, some items were modified in the qualitative content validation phase and 23 items were omitted due to their high similarities with other items. Additionally, the item “How far does the school dean and deputies try to empower/develop the staff?” was added. The shared governance instrument was prepared with 89 items to undergo quantitative content validation. Twelve items were deleted in the CVR survey due to a CVR of less than 0.60 [33]. The cut-off point was set at 0.80 to determine CVI. Moreover, SCVI was obtained as 0.910 with the mean approach that was appropriate.

The inter-rater consensus index was excellent or good for most items. Finally, the instrument entered the construct validation stage with 77 items. The primary reliability of the instrument was estimated before construct validation. Cronbach’s alpha of the shared governance instrument with 77 items was 0.975 that was acceptable. Furthermore, the results of the item analysis performed in this phase, the correlation among the items, between each item and the whole instrument were calculated. The correlation coefficient of 7 items was less than 0.30. Thus, the reliability coefficient was estimated even in the exclusion of the said items.

**Fig. 2:**
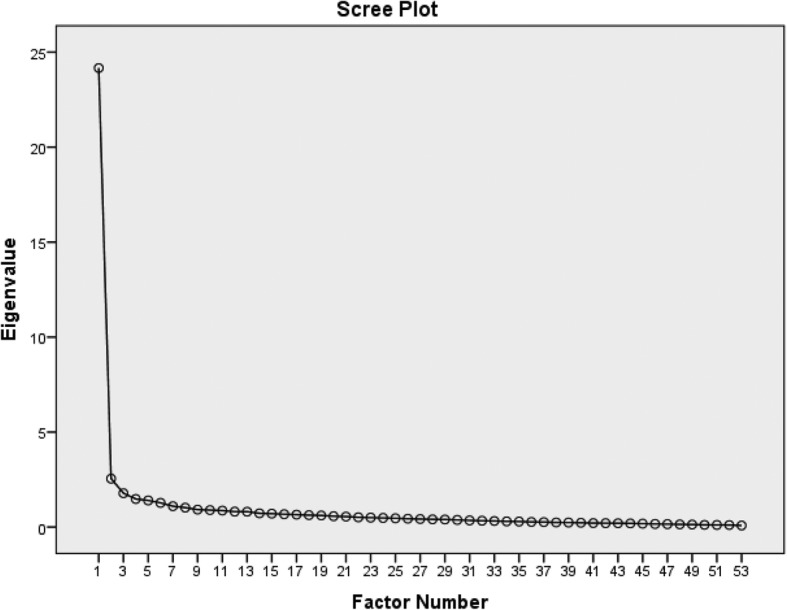
The scree plot of the shared governance instrument

Although Cronbach’s alpha of all the related categories reduced after omission of the items, all of these 7 items were deleted by the research team due to the increase of total reliability of the instrument to 0.977. The age of 194 participants in the construct validation ranged between 29 and 61 years. The mean work experience of individuals was less than 20 years. Most participants were female, held a PhD degree in nursing, worked as assistant professors and most had not taken part in management courses and workshops. The KMO was estimated at 0.953 indicating a sufficient number of samples in factor analysis. The statistical significance of Bartlett’s sphericity test (*P* = 0.000) suggested proper conditions of factor analysis. In the next stage, the factors were extracted and the variables with high correlation were put in a class or factor. In addition, this study used a scree plot (Fig. 2) and cumulative variance percentage determined by the extracted factors.

The results of factor analysis with Promax rotation demonstrated a bi-factorial structure with a total variance of 78.6% for 52 items of shared governance instrument. Eighteen items were omitted in this stage and the number of items was reduced to 52. Thus, 44.6% of the common variance was accounted for by the first factor and 3.95% by the second factor (Table 2).

**Table 2:** Factors extracted by factor analysis by promax rotation and factor loadings of items of each factor in shared governance instrument

Cumulative variance percentage	Item no.	Item	First factor	Second Factor
First factor: Shared governance atmosphere and culture Variance percentage = 44.618	1	How much reciprocal confidence exists between school “dean and deputies” and faculty members?	0.9	
	2	How much is the behavior of school dean and deputies associated with affability and conciliation at the time of trouble for educational ward managers?	0.875	
	3	How much formal and organised communication is there between faculty members and school dean and deputies?	0.856	
	4	How much effort is made by school dean and deputies to empower the staff?	0.825	
	5	How much effort is made by school dean and deputies to empower the faculty members?	0.800	
	6	How much importance is attached to criticisms and recommendations received from criticisms box by the school dean and deputies?	0.779	
	7	How much is the performance of school dean and deputies in line with school goals?	0.776	
	8	How much are school dean and deputies competent in managing conflict/approaching opposite opinions?	0.773	
	9	How much reciprocal respect is there between and among the beneficiary groups in the school?	0.773	
	10	How much collaboration and coordination is there between all beneficiaries, especially between faculty members and school dean and deputies?	0.756	
	11	How much feeling of equality is there between school staff and managers?	0.745	
	12	How much effort is made by school dean and deputies to empower students?	0.726	
	13	What is the rate of application of informal and friendly rapport that supports sharing by school dean and deputies?	0.724	
	14	How much effort is made by school dean, deputies, and faculty members to clarify the reasons of their decisions about others?	0.714	
	15	How much distribution of power exists in the school?	0.713	
	16	How far are school dean and deputies responsible for shared decision-makings?	0.710	
	17	How humanly are the relations among beneficiaries?	0.637	
	18	How far have school dean and deputies been able to align individual goals of beneficiaries with organisational goals?	0.630	
	19	How far are programs by faculty members for managing school affairs celebrated and supported by school dean and deputies?	0.630	
	20	How far is contribution of school dean and deputies based on staff capabilities?	0.618	
	21	How much do school dean and deputies verbally and practically propagate the contributory culture in the school?	0.617	
	22	How much is the behavior of educational ward managers associated with affability, conciliation, and reciprocal understanding at the times of trouble?	0.581	
	23	How far do school dean and deputies cooperate with affiliated hospitals and healthcare centers to investigate educational, research, and managerial problems of clinical setting?	0.527	
	24	How much free space is there for faculty members to pose and discuss their scientific questions?	0.510	
	25	How much importance is attached to compatibility of affairs with environmental changes (social, technological, economical, and political) by higher order and intermediary managers for shared management of school affairs?	0.510	
	26	How much transfer of power and delegation is there for implementing shared programs in school?	0.483	
	27	How much importance is attached equally to agreeable and disagreeable opinions on a specific issue in decision-making sessions?	0.475	
	28	How much ability do school dean and deputies have to adjust centralized rules to ease faculty members’ contribution?	0.470	
	29	How much spiritual award is devoted to shared activities of faculty members at school?	0.439	
	30	How regularly do intragroup committees meet on the basis of discipline and protocols?	0.439	
	31	How far do faculty members play a role in assessment of dean and deputies’ performance?	0.429	
Second factor: Infrastructural prerequisites Variance percentage = 3.950	32	How far do instructors and students set goals at work collaboratively?		0.755
	33	How much time do mangers/educational departments agents spend on consultation with faculty members, before their vote on issues in councils and meetings?		0.751
	34	How far do faculty members have access to the information for shared decision-makings?		0.737
	35	How much do “mean work hours/due credit hours per month of faculty members” pave the way for shared management of affairs?		0.707
	36	How much importance do outsider assessors attach to implementing shared governance in periodical assessments of the school?		0.700
	37	How much material award is devoted to faculty members’ shared activities in school?		0.649
	38	How far do rules and regulations (educational, cultural, research, and administrative) facilitate performance of faculty members’ duties?		0.608
	39	How far do expectations of educational wards managers from school faculty members guide them toward sharing?		0.579
	40	How far do the physical shape and building of school (decoration of classroom seats and desks, meeting rooms, professors’ rooms, managers’ rooms, etc.) facilitate sharing?		0.568
	41	How quickly do faculty members inform educational wards mangers about their decisions?		0.566
	42	How much are students allowed to contribute to ward/department decision-makings?		0.544
	43	How far do faculty members play a role in selecting their representatives in managerial committees, management board, or extra organisational sessions?		0.542
	44	How far are protocols and guidelines provided by the university based on contribution of faculty members to managing school affairs?		0.539
	45	How far have educational wards managers been able to align faculty members’ individual goals with organisational goals?		0.516
	46	How much do faculty members or their representatives contribute to managerial decision-makings like setting goals, strategic planning, budgeting, etc.?		0.441
	47	What degree of sharing or contributory spirit exists in faculty members?		0.424
	48	How far are faculty members responsible in shared decision-makings?		0.423
	49	How much welfare facilities (nursery, transportation, self-service, publication office, etc.) are available to faculty members at school?		0.419
	50	How far are educational wards managers responsible for shared decision-makings of ward/department members?		0.410
	51	How much feeling of belonging and dependence do faculty members have toward school?		0.400
	52	How much independence do faculty members enjoy in planning and revising of educational syllabus/curriculum?		0.400

Labeling of factors was completed through a mental, theoretical, and deductive process by considering the dimensions identified during the qualitative stage of the study under the guidance and consultation of the research team. After completing the factor analysis and omission of some items, reliability was established again via internal consistency on 194 participants. To examine the internal consistency of the whole instrument, Cronbach’s alpha, Omega index, and split-half reliability via the usual and odd/even method were used. The results suggested a high internal consistency of the whole questionnaire and all factors (Table 3).

**Table 3:** Internal consistency of shared governance instrument after factor analysis

Factor number	Factor name	Number of items	Cronbach’s alpha	Omega	Ordinary Split-half	Odd/even split half
1	Shared atmosphere and culture	31	0.972	0.802		
2	Infrastructural prerequisites	21	0.930	0.716		
Total			0.981	0.805	0.904	0.968

The test-retest method was used to determine instrument stability, with an ICC of 0.911 suggesting high consistency of the instrument with SEM of 10.43 (Table 4).

**Table 4:** Examination of consistency of shared governance instrument

Factor	Mean (SD)	ICC	CI (95%)	SEM
1	80.844 (23.941)	0.897	(0.792–0.949)	7.683
2	56.796 (15.192)	0.908	(0.815–0.955)	4.607
Total	137.963 (34.963)	0.911	(0.821–0.956)	10.43

The questionnaire items were weighted using the formula given above. By comparing the mean weights obtained with both methods of weighted and non-weighted items, paired t-test showed a significant difference between the two methods (*P* = 0.000, T = 48.81). Thus, to interpret the results obtained from the scale, it is better to estimate the weighted Likert values. Ultimately, the shared governance feasibility instrument was developed with 52 items and 2 factors of “shared atmosphere and culture” and “infrastructural prerequisites” (supplement 1).

## Discussion

This study developed and validated a shared governance feasibility instrument. Since any measurement is valid in so far as it measures what it is intended to measure [34], in this study the validation process began with psychometric face validation. The studies abroad on shared governance instrument development have either not discussed face validity [11, 13, 18] or if they have reported it in papers [12], it seems that they have all used only qualitative face validity, as they did not report quantitative estimation of face validity and item impact index. For example, in the study by Zhang, 10 items were omitted in the qualitative face validation stage. In the next phase, the content validity of the instrument was investigated [12]. The studies abroad on implementation of instruments in examining shared governance have either not reported content validation or if reported, they have used an adjusted form of AAUP instrument of shared governance [11, 12, 20]. This study used EFA with Promax rotation. Factor analysis extracted the structures of the two factors that had acceptable validity and reliability so that the two factors accounted for 78.6% of the total variance.

The first factor, i.e., “shared atmosphere and culture”, is in line with the “overall climate for shared governance” that is one characteristic of Ramo’s shared governance indicators [8, 10, 11, 19]. Indeed, the first three items of the first factor, i.e., reciprocal confidence [18, 19, 35], conciliation, reciprocal understanding [36], and communication [11, 19, 37] are in line with the “overall climate for shared governance” index from Ramo’s shared governance indicators. The second factor was “infrastructural prerequisites” although there was no index with this label, in review of literature or in Ramo’s shared governance indicators. There are some items of this factor that are in line with studies that have mentioned the following items as necessary for the successful implementation of shared governance: maintaining acceptable workload for faculty members, having sufficient time for sharing [19], accessibility of information, support, and resources for the staff [38, 39], having a positive feeling toward the work environment and moving towards organisational goals [39]. A quantitative study used construct validation of EFA type to adjust the parts of National Survey of Community College Leaders (NSCCL) instrument pertaining to shared governance and satisfaction. In the present study, the shared governance instrument showed proper internal consistency during investigation of reliability in the first stage and during investigation of internal consistency in the second stage. Each item showed a high correlation with the whole instrument, indicating that all items measure the same construct. Test-retest was used to explore consistency (stability) of the instrument (Cronbach’s alpha) whereas the shared governance instrument used in Finnell (2014) did not deal with this aspect of reliability and has sufficed to examining internal consistency [39].

The shared governance instrument entered the item weighting stage with 52 items. The weighting of items can provide more accurate results [40]. Nevertheless, none of the shared governance instruments abroad has weighed the items [11, 12, 20, 39]. In the present study, the mean scores of various factors in the instrument were significantly different before and after weighting.

Although most studies on shared governance have used instruments derived from AAUPISG (The American Association of University Professors Indicators of Sound (Shared Governance), the instrument merely reflects some concepts of the AAUP’s 1966 Statement on Government of Colleges and Universities and does not deal with feasibility. Some strong points of the instrument developed in this study include: extraction of items with an inductive-deductive approach (review of literature and interview with faculty members) whereas none of the present instruments are based on faculty members’ experiences [11, 12]. Finnell has recommended that future studies focus on a qualitative study using interviews with individuals to better understand the attitudes of managers [39]. Eventually, the findings revealed that the 52-item “shared governance instrument” was acceptable in validity and reliability with respect to the two factors.

### Limitations

One limitation of the present study was difficult access to higher order, intermediary managers of medical universities and their limited free time for interviews in the qualitative stage of the study. This was due to their highly busy condition, which was time-consuming process for interview. Another limitation was the difficulty in accessing to faculty members at all nursing schools, of major medical universities in Iran, to allow completion the questionnaires. The researchers followed up the deficient questionnaires frequently in order to decrease this limitation as much as possible.

## Conclusion

To answer the first question of this study (what is meaning of shared governance concept in nursing schools?) hybrid content analysis showed that shared governance is like “several souls in one body” in the Iranian cultural context with its emphasis on all aspects of organisational contribution, ethics, and spirituality. Managers of higher education and nursing schools should pay due attention to all aspects of shared governance, especially spirituality in managing their organisations. Additionally, answering the second question of this research (does the designed tool have optimal validity and reliability?), the findings showed that the instrument has acceptable validity and reliability, and confirmed construct validity in the two-factor model.

This instrument can be used by nursing school managers to measure the feasibility of the shared governance in their organisation. Managers and policy-makers at the level of the related ministry of health, medical universities and affiliated nursing schools are advised to apply the results of this study to prepare for revising their centralised management policies and move forward to decentralised and independence. They should make decisions at the macroscopic and microscopic levels to increase the contribution of all beneficiaries and interested parties. It appears that providing infrastructural prerequisites for implementing shared governance would not only lead to the use of intelligence, capacities of beneficiaries and faster movement toward achieving organisational goals, but also serve as a practical guide for institutionalising student participation in various professional and social fields in future.

## Implications for nursing management

The results demonstrated that, except one item that pertained to faculty clinicians, all items of the developed shared governance instrument are designed in a way that provides highly clear attitude of implementing shared governance to policy-makers, higher education centers, and managers. It is also hoped that this study and the developed instrument can serve as guide for the feasibility of implementing shared governance to assess management styles and performance in higher education centers.

## Supplementary information

**Additional file 1.** Shared governance feasibility questionnaire.

## Data Availability

All data generated or analysed during this study are included in this published article and its supplementary file (Shared Governance Feasibility Questionnaire).

## Abbreviations

AAUP: American Association of University Professors
AAUPISG: The American Association of University Professors’ Indicators of Sound Governance
CVR: Content validity Ratio
CVI: Content validity Index
SEM: Standard Error of Measurement
EFA: Exploratory Factor Analysis
KMO: Kaiser-Meyer-Olkin
ICC: Intra class correlation
