# Supplementary material for: Development and validation of the shared governance feasibility instrument in nursing schools in Iran
Source: BMC Nurs. 2020 May 20;19:42. doi: 10.1186/s12912-020-00433-x (PMC7238626; doi:10.1186/s12912-020-00433-x)
Supplement: Supplementary file 1 — Additional file 1. Shared governance feasibility questionnaire. [file 12912_2020_433_MOESM1_ESM.docx]

| Shared Governance Feasibility Questionnaire | | |
| --- | --- | --- |
| **Factor** | **no** | **Items** |
| Shared atmosphere and culture | 1 | How much reciprocal confidence exists between school “dean and deputies” and faculty members? |
|  | 2 | How much is the behavior of school dean and deputies associated with affability and conciliation at the time of trouble for educational ward managers? |
|  | 3 | How much formal and organised communication is there between faculty members and school dean and deputies? |
|  | 4 | How much effort is made by school dean and deputies to empower the staff? |
|  | 5 | How much effort is made by school dean and deputies to empower the faculty members? |
|  | 6 | How much importance is attached to criticisms and recommendations received from criticisms box by the school dean and deputies? |
|  | 7 | How much is the performance of school dean and deputies in line with school goals? |
|  | 8 | How much are school dean and deputies competent in managing conflict/approaching opposite opinions? |
|  | 9 | How much reciprocal respect is there between and among the beneficiary groups in the school? |
|  | 10 | How much collaboration and coordination is there between all beneficiaries, especially between faculty members and school dean and deputies? |
|  | 11 | How much feeling of equality is there between school staff and managers? |
|  | 12 | How much effort is made by school dean and deputies to empower students? |
|  | 13 | What is the rate of application of informal and friendly rapport that supports sharing by school dean and deputies? |
|  | 14 | How much effort is made by school dean, deputies, and faculty members to clarify the reasons of their decisions about others? |
|  | 15 | How much distribution of power exists in the school? |
|  | 16 | How far are school dean and deputies responsible for shared decision-makings? |
|  | 17 | How humanly are the relations among beneficiaries? |
|  | 18 | How far have school dean and deputies been able to align individual goals of beneficiaries with organisational goals? |
|  | 19 | How far are programs by faculty members for managing school affairs celebrated and supported by school dean and deputies? |
|  | 20 | How far is contribution of school dean and deputies based on staff capabilities? |
|  | 21 | How much do school dean and deputies verbally and practically propagate the contributory culture in the school? |
|  | 22 | How much is the behavior of educational ward managers associated with affability, conciliation, and reciprocal understanding at the times of trouble? |
|  | 23 | How far do school dean and deputies cooperate with affiliated hospitals and healthcare centers to investigate educational, research, and managerial problems of clinical setting? |
|  | 24 | How much free space is there for faculty members to pose and discuss their scientific questions? |
|  | 25 | How much importance is attached to compatibility of affairs with environmental changes (social, technological, economical, and political) by higher order and intermediary managers for shared management of school affairs? |
|  | 26 | How much transfer of power and delegation is there for implementing shared programs in school? |
|  | 27 | How much importance is attached equally to agreeable and disagreeable opinions on a specific issue in decision-making sessions? |
|  | 28 | How much ability do school dean and deputies have to adjust centralised rules to ease faculty members’ contribution? |
|  | 29 | How much spiritual award is devoted to shared activities of faculty members at school? |
|  | 30 | How regularly do intragroup committees meet on the basis of discipline and protocols? |
|  | 31 | How far do faculty members play a role in assessment of dean and deputies’ performance? |
| Infrastructural prerequisites | 32 | How far do instructors and students set goals at work collaboratively? |
|  | 33 | How much time do mangers/educational departments agents spend on consultation with faculty members, before their vote on issues in councils and meetings? |
|  | 34 | How far do faculty members have access to the information for shared decision-makings? |
|  | 35 | How much do “mean work hours/due credit hours per month of faculty members” pave the way for shared management of affairs? |
|  | 36 | How much importance do outsider assessors attach to implementing shared governance in periodical assessments of the school? |
|  | 37 | How much material award is devoted to faculty members’ shared activities in school? |
|  | 38 | How far do rules and regulations (educational, cultural, research, and administrative) facilitate performance of faculty members’ duties? |
|  | 39 | How far do expectations of educational wards managers from school faculty members guide them toward sharing? |
|  | 40 | How far do the physical shape and building of school (decoration of classroom seats and desks, meeting rooms, professors’ rooms, managers’ rooms, etc.) facilitate sharing? |
|  | 41 | How quickly do faculty members inform educational wards mangers about their decisions? |
|  | 42 | How much are students allowed to contribute to ward/department decision-makings? |
|  | 43 | How far do faculty members play a role in selecting their representatives in managerial committees, management board, or extra organisational sessions? |
|  | 44 | How far are protocols and guidelines provided by the university based on contribution of faculty members to managing school affairs? |
|  | 45 | How far have educational wards managers been able to align faculty members’ individual goals with organisational goals? |
|  | 46 | How much do faculty members or their representatives contribute to managerial decision-makings like setting goals, strategic planning, budgeting, etc.? |
|  | 47 | What degree of sharing or contributory spirit exists in faculty members? |
|  | 48 | How far are faculty members responsible in shared decision-makings? |
|  | 49 | How much welfare facilities (nursery, transportation, self-service, publication office, etc.) are available to faculty members at school? |
|  | 50 | How far are educational wards managers responsible for shared decision-makings of ward/department members? |
|  | 51 | How much feeling of belonging and dependence do faculty members have toward school? |
|  | 52 | How much independence do faculty members enjoy in planning and revising of educational syllabus/curriculum? |
